# Regulation of Deubiquitinating Enzymes by Post-Translational Modifications

**DOI:** 10.3390/ijms21114028

**Published:** 2020-06-04

**Authors:** Tanuza Das, Sang Chul Shin, Eun Joo Song, Eunice EunKyeong Kim

**Affiliations:** 1 Biomedical Research Institute, Korea Institute of Science and Technology, Seoul 02792, Korea; d17606@kist.re.kr (T.D.); scshin84@kist.re.kr (S.C.S.); 2 Graduate School of Pharmaceutical Sciences and College of Pharmacy, Ewha Womans University, Seoul 03760, Korea; esong@ewha.ac.kr

**Keywords:** post-translational modification (PTM), deubiquitinase (DUB), deubiquitinating enzyme, activity, localization, interaction, disease

## Abstract

Ubiquitination and deubiquitination play a critical role in all aspects of cellular processes, and the enzymes involved are tightly regulated by multiple factors including posttranslational modifications like most other proteins. Dysfunction or misregulation of these enzymes could have dramatic physiological consequences, sometimes leading to diseases. Therefore, it is important to have a clear understanding of these regulatory processes. Here, we have reviewed the posttranslational modifications of deubiquitinating enzymes and their consequences on the catalytic activity, stability, abundance, localization, and interaction with the partner proteins.

## 1. Introduction

Ubiquitination, the covalent attachment of 76 amino acid polypeptide ubiquitin (Ub) to a substrate protein, is a reversible post-translational modification (PTM) process involved in the regulation of most cellular processes. Ubiquitination affects the target proteins in many ways: marks them for degradation via the proteasome, alters their specific location in the cell, affects their activity or stability, and promotes or prevents interactions with their partners. As such, the aberrations in the system, directly or indirectly, result in the pathogenesis leading to diseases including cancers, inflammatory, and neurodegenerative disorders. Ubiquitin-conjugation to a substrate protein is performed sequentially by a cascade of enzymes called E1, E2, and E3, and there are about 700 enzymes (~40 E2 and ~600 E3) to generate the ubiquitinated substrate [1,2]. Not surprisingly, ubiquitination can be reversed by cleaving Ub from the substrate protein to terminate the signal. This process is called deubiquitination (or deubiquitylation) which is carried out by a group of protease named deubiquitinating (or deubiquitylating) enzymes or simply DUBs [3]. The human genome encodes approximately 80 DUBs that are predicted to be actively opposing the function of E3 ligases. Therefore, ubiquitination is quite similar to protein phosphorylation in being reversible and both are mediated by a much larger abundance of enzymes for the forward reaction (kinases and ligases) than the reverse reaction (phosphatases and DUBs) [4].

**Figure 1:**
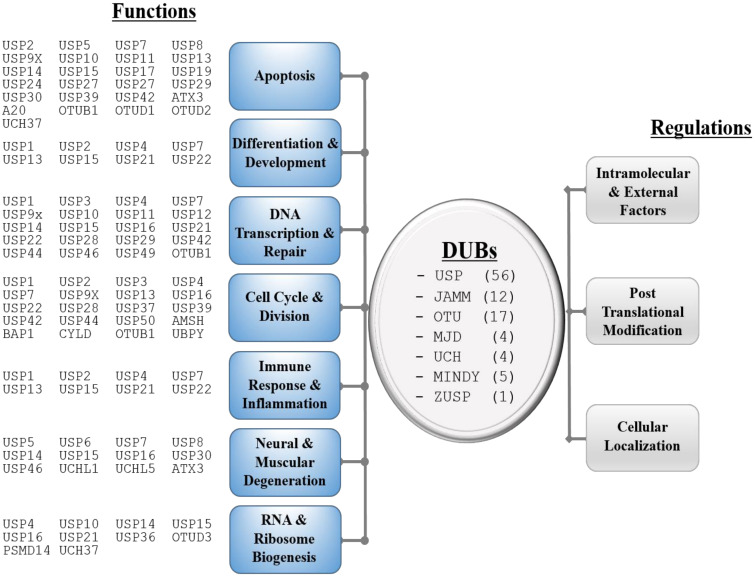
Diverse functions and regulations of deubiquitinating (or deubiquitylating) enzymes (DUBs). DUBs are grouped into seven categories based on their functions.

DUBs can be sub-grouped into seven evolutionarily conserved families: ubiquitin-specific proteases (USPs), JAB1/MPN/Mov34 metalloenzyme (JAMM; also known as MPN), ovarian tumor proteases (OTUs), Josephin and JAB1/MPN^+^(MJP), ubiquitin C-terminal hydrolases (UCHs), and two recently discovered MIU-containing novel DUB (MINDY) and zinc finger-containing ubiquitin peptidase 1 (ZUP1) as shown in Figure 1. DUBs can regulate Ub-dependent metabolic pathways in several ways: (1) by processing linear polyUb precursors into single Ub molecules, (2) by recycling Ub to generate free Ub that may participate in further Ub conjugation process, (3) by preventing E3 ligases mediated Ub conjugation, or (4) by removing Ub from ubiquitinated substrates [5,6]. Ub balance is achieved via all these aforementioned processes, and thus, plays major roles in many essential biological processes such as cell cycle and division, DNA transcription and repair, differentiation and development, immune response, neural and muscular degeneration, apoptosis, and RNA and ribosomal biogenesis as shown in Figure 1. Dysfunction of certain DUBs could have dramatic physiological consequences including diseases, and their involvement in disease has triggered drug discovery efforts in recent years [7]. However, the full extent of the role of DUBs in diseases is yet to be unraveled.

As DUBs are involved in many important and critical cellular functions, cells adopt several strategies to regulate their activities to take place at the right sites at the right moments [8]. As such, in addition to being regulated at the transcription level, DUBs themselves undergo multiple layers of regulation including PTMs [9,10,11]. As shown in Figure 1, the known regulatory mechanisms for DUBs to date include regulation by intramolecular or external factors, allosteric interactions, subcellular localization, and by different PTMs, with some being critical for their functions. Many DUBs contain other domains and/or structural motifs besides the catalytic domain, and these non-catalytic regions/domains enable the interaction with target proteins as well as proteins that regulate their catalytic activity. A single PTM sometimes can re-direct the entire downstream signal, and the aberrant state of PTMs are sometimes implicated in human diseases. Therefore, a good understanding of the underlying mechanism of these regulations is necessary, especially considering a specific DUB as a target for the development of therapeutic agents. With recent advancements in biology, there has been an accumulation of data on PTMs on DUBs. In this review, we focused on the known PTMs of most commonly studied DUBs and their functional and regulatory effects in cells.

## 2. Post-Translational Modifications of DUBs

The reports on the regulation of DUBs by various factors including PTMs have not been extensive, but some have enlightened the extent and elegance of such regulation [10]. In some cases, the same PTM can result in different functional outcomes, while a combination of more than one PTMs is necessary. Crosstalk between PTMs, prominently between phosphorylation and ubiquitination, are also possible, and it can act either positively or negatively in both directions, as noted earlier [12]. However, this is beyond the scope of this review. Thus far, phosphorylation, ubiquitination, and SUMOylation are the recognized PTMs, and Table 1 lists the known PTMs of various DUBs and their effects. Below, we describe our current knowledge of DUB regulation by PTMs and the effect on their activity, abundance, cellular localization, and interactions with partner proteins.

### 2.1. PTMs Regulating the Catalytic Activity of DUBs

**Figure 2:**
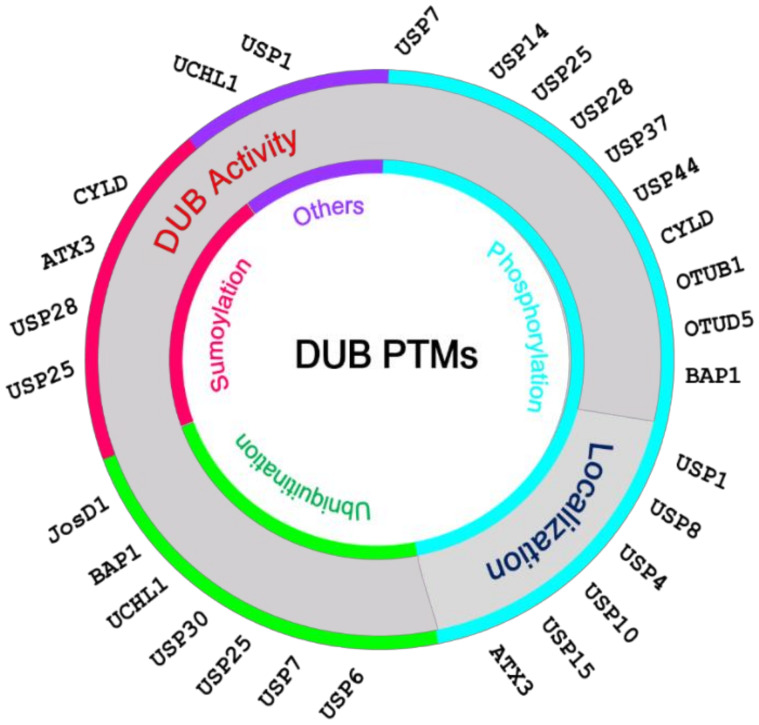
Post-translational modifications (PTMs)-mediated regulations on DUB.

Regulation of the catalytic activity by several PTMs has been reported for certain DUBs, with the protein phosphorylation being the most frequent thus far (Table 1 and Figure 2). This is in line with the findings on each PTM complied in the Swiss-Prot database, i.e., phosphorylation is seen more than twice of all other PTMs together [13]. In contrast to many other proteases that are translated as inactive precursors, such as caspases, DUBs are usually formed as an active enzyme. However, in some cases, the catalytic activity of DUBs is achieved only when it is incorporated into a multi-component complex. For example, there are indeed three DUBs directly associated with proteasome: UCHL5, USP14 (also known as Ubp6 in yeast), and RPN11. In the case of USP14, the activity enhances as high as 800-fold upon association with the proteasome through its ubiquitin-like (UBL) domain [14]. The crystal structures of USP14 in isolation and Ub-aldehyde complex show that the two loops near the active site, called blocking loop 1 (BL1) and BL2 block the active site in isolation, while they take up different conformations allowing the active site Cys114 to cleave Ub chains from a substrate. A recent study showed that the phosphorylation by AKT on the highly conserved Ser432 of USP14 enhances the DUB activity in proteasome in vitro and cells, and this is critical in regulating proteasome activity and, consequently, global protein degradation [15]. Ser432 is located on the BL2 close to the highly negatively charged patch formed by Glu188, Asp199, and Glu202, and phosphorylation of Ser432 is thought to induce conformational change to promote activation of the active site of USP14.

**Figure 3:**
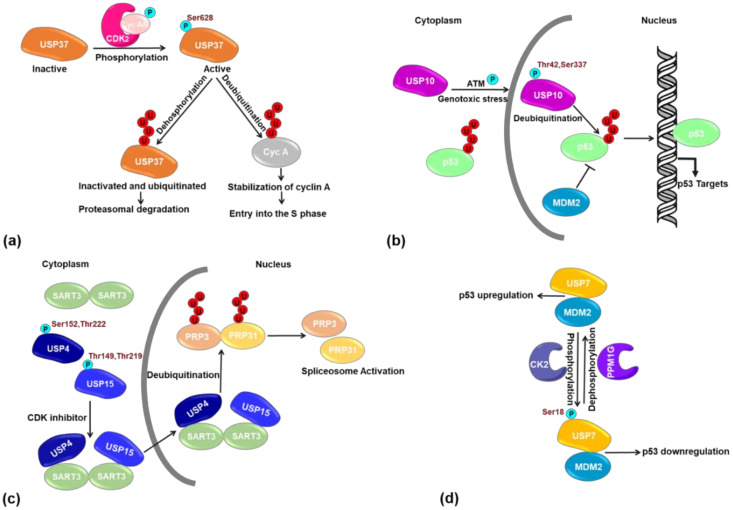
Examples of phosphorylation on various DUBs. (**a**) Phosphorylation of USP37 in G1/S triggers its full DUB activity. Phospho-USP37 stabilizes cyclin A by removing degradative polyUb, whereas dephosphorylated USP37 can be ubiquitinated and undergoes proteasomal degradation in late mitosis [17]. (**b**) Phosphorylated USP10 translocates into the nucleus where it deubiquitinates p53 and inhibits Mdm2-induced p53 degradation [49]. (**c**) USP15 and USP4 dephosphorylation lead to their interaction with SART3, which in turn allows DUB translocation into the nucleus and the regulation of spliceosomal function [92]. (**d**) CK2-mediated phosphorylation stabilizes USP7, resulting in Mdm2 stabilization and p53 downregulation. USP7 dephosphorylation by PPM1G destabilizes USP7 resulting in Mdm2 degradation and upregulation of p53 [95].

Several DUBs have been identified as critical regulators of mitosis, and some are reported to undergo further regulation by PTM, thereby making the mitosis process more complex and dynamic [16]. For example, the catalytic activity of USP37, a cell cycle regulating DUB, is regulated by phosphorylation. USP37 binds to APC/C adaptor protein CDH1 in G1/S and removes degradative polyUb from the APC^CDH1^ substrate cyclin A. USP37-mediated deubiquitination and stabilization of cyclin A enable entry into the S phase. In G1/S, Ser628 of USP37 is phosphorylated by either CDK2/cyclin E or CDK2/cyclin A, and this triggers USP37 full DUB activity. However, in late mitosis, USP37 is inactivated by loss of phosphorylation due to the inactivation of the kinase CDK2 (Figure 3a). This inactive USP37 becomes a substrate for the E3 ubiquitin ligase APC^CDH1^ and undergoes proteasomal degradation by Lys11-linked polyubiquitination. The point mutant USP37-S628A had a considerably lower DUB activity, but how the phosphorylation at Ser628 promotes USP37 DUB activity is not yet well understood, although it has been suggested that the phosphorylation induces USP37 binding to its substrates by rearranging the ubiquitin-interacting motifs [17]. Phosphorylation-dependent activation on another DUB, USP44, is also critical for cell cycle regulation by maintaining the spindle checkpoint assembly. The stabilization of the APC inhibitory Mad2-Cdc20 complex both in vitro and in vivo pointed to mitotic cyclin-dependent kinases (CDKs) or spindle checkpoint kinases being likely involved in USP44 phosphorylation [18]. Besides phosphorylation, USP44 undergoes both Lys48- and Lys63-linked poly-ubiquitination, which regulates its proteasomal degradation and stability [19]. Another DUB USP8 phosphorylation in interphase inhibits its DUB activity either directly or through the recruitment of 14-3-3 protein family members [20]. This phosphorylation is lost in mitosis, which results in the increased activity of USP8, probably by recruiting to the mid-body during cytokinesis [21,22]. Phosphorylation of USP8 at additional Ser and Tyr residues has been reported to regulate its function although the exact outcomes are not yet defined [23]. Additionally, USP8 phosphorylation is also important for the regulation of ciliogenesis in the dividing cells. USP8 phosphorylation on Tyr717 and Tyr810 by EGFR kinase elevates its activity and then activates an inhibitory mechanism of ciliogenesis, i.e., EGF receptor kinase suppresses ciliogenesis through activation of USP8 deubiquitinase [24].

In the adaptive immune response, USP9X phosphorylation is required for the activation of lymphocytes. During priming of T lymphocyte, USP9X removes the inhibitory mono-ubiquitination from the protein kinase ZAP-70. The T-cell receptor (TCR)-dependent phosphorylation of USP9X at Ser1600 is necessary for its maximal catalytic activity, and this is also required for enhancing PKCβ kinase activity in B-lymphocytes. The TCR phosphoproteome mass spectrometric analysis revealed that Ser1600, which lies within the ubiquitin carboxyl-terminal hydrolase (UCH) domain conferring USP9X catalytic activity, is highly conserved among multiple species [25]. Therefore, activation of lymphocytes by USP9X phosphorylation might be an attractive aspect to harness the immune system for therapeutic benefit. Phosphorylation of OTUD5 (also known as DUBA) at Ser177 is both necessary and sufficient to activate the enzyme which is a negative regulator of type I interferon [26]. The crystal structure of the Ub-aldehyde complex with phosphorylated OTUD5 reveals a remarkable interaction between the phosphorylated Ser177 of OTUD5 and the guanidinium moiety of Arg74 of the bound ubiquitin showing that the phosphorylation is essential for the DUB activity [27]. A recent study using NMR and enzymatic kinetics of different forms of OTUD5 confirmed that only the phosphorylated OTUD5 is important for the activity [28].

Phosphorylation of ubiquitin carboxyl-terminal hydrolase CYLD, a tumor suppressor, and an important player in the host defense mechanism against bacterial infection, at Ser418 by the inhibitor of nuclear factor-κB kinase (IKKγ), impairs its DUB activity. This, in turn, contributes to the activation of JNK and IKK, thereby positively regulate nuclear factor-κB (NF-κB) activation [29]. Another NF-κB inhibitory DUB, OTULIN inhibits Ser418 phosphorylation of CYLD through a LUBAC-dependent mechanism, which sustains its catalytic activity, while hyperphosphorylation of OUTLIN at Tyr56 regulates necroptosis by modulating RIPK1 ubiquitin dynamics [30]. A recent study showed that CYLD phosphorylation is elevated in transformed cells and inhibition of this phosphorylation by IKK inhibitors triggers apoptosis, suggesting CYLD as a novel therapeutic target for adult T-cell leukemia [31]. A20, unlike other DUBs, contains both DUB and E3 ligase domains. The DUB activity of the N-terminal OTU domain is essential for NF-kB signaling factors. In particular, phosphorylation of A20 at Ser381 by kinase IKKβ causes an increase in DUB activity towards NF-kappa-B essential modulator (NEMO) [32]. Recently identified three variants of A20 showed a varying degree of phosphorylation at Ser381 by IKKβ (T108A/I207L > I325N > C243Y), and interestingly, the graded phosphorylation status consequently caused a graded reduction in A20 function and control of NF-κB [33].

Besides phosphorylation, ubiquitination comprises another mode of regulation for DUB activity. For instance, the activity of UCHL1, an abundant DUB in the brain, is negatively regulated by mono-ubiquitination [34] and its dysfunction is implicated in several neurodegenerative diseases including Parkinson’s disease [35,36]. Since the identified ubiquitination sites (Lys4, Lys65, Lys71, or Lys157) are near the active site, it is thought ubiquitination at these sites prevents UCHL1 association with the ubiquitinated substrate. However, the physiological substrate of UCHL1 and the E3 ligase have not been identified thus far. It was also noted that UCHL1 deubiquitinates itself, although the role of autoregulation is not clear. On the other hand, the ubiquitination of ataxin-3 (ATX3) at Lys117 is reported to enhance its DUB activity [37]. ATX3 is a DUB, toxic gain of function to the CAG (polyQ) expansion lead to cerebrospinal ataxia-3 (also known as Machado-Joseph disease), an autosomal dominant neurodegenerative disorder. Additionally, JosD1 cleaves the Ub chains only after it is mono-ubiquitinated in vitro, although the ubiquitination site has not yet been identified. JosD2, which shares high sequence homology with JosD12, cleaves the Ub chain without ubiquitination. JosD2 localizes to the cytoplasm whereas JosD1 preferentially localizes to the plasma membrane particularly when mono-ubiquitinated [38].

Additionally, modifications by ubiquitin-like molecules, such as SUMO, have been reported to regulate the DUB activity. For example, the DUB activity of both USP25 [39] and USP28 [40] is impaired upon SUMOylation. Lys99 and Lys141 were identified for USP25, while Lys99 was identified as the major SUMOylation for USP28. All three are located either in the ubiquitin interacting motif (UIM) region or at the beginning of the UIM regions located at the N-terminal preceding to the catalytic domain, and it was suggested that SUMOylation at these sites most likely block the Ub binding. Interestingly, the two DUBs are evolutionarily related by an identical overall domain architecture, but are functionally non-redundant: USP28 stabilizes c-MYC and other nuclear proteins, while USP25 regulates inflammatory TRAF signaling. It should be noted that Lys99 of USP25 could be mono-ubiquitinated, enhancing its catalytic activity and substrate recognition resulting in potentially an opposite functional outcome from SUMOylation [41]. Recently, the catalytic domain of both USP25 and USP28 was shown to form a dimer [42,43]. Surprisingly, USP25, but not USP28, is regulated by further oligomerization, i.e., the DUB activity of USP25 is auto-inhibited by tetramerization through sequences inserted into their catalytic domains [44].

Oxidation, a non-enzymatic addition in vitro is important for DUBs, since most of them are cysteine proteases. They share a common mechanism that involves a nucleophilic attack of the catalytic Cys on the substrate carbonyl carbon to form a thiol acyl intermediate. This thio-acyl then reacts with water to fully hydrolyze the amide bond and remove the Ub moiety from the substrate. Thus, these DUBs are sensitive to the oxidative environment, e.g., reactive oxygen species that might change—SH of the catalytic Cys to -SOH, -SO_2_H, or -SO_3_H, thereby reducing the DUB activity. Indeed, USP1 was shown to reversibly inactivated by oxidation in its catalytic Cys [45,46,47]. The crystal structures of four different oxidation states of A20 revealed that the reversible form of A20 oxidation is a cysteine sulfenic acid intermediate, which is stabilized by the architecture of the catalytic center [45,46,47].

### 2.2. PTMs Regulating the Subcellular Localization of DUBs

Subcellular localization is another important factor in the regulation of DUB activity and substrate availability since the activity and substrate availability might be well be determined by the subcellular localization. A systematic survey of 66 DUBs with a green fluorescent protein in HeLa cells revealed that they are distributed all over the cell. A significant number of DUBs are accumulated in the nucleus or cytoplasm; many are cytonuclear, while some others show specific association with a variety of defined structures, including the nucleolus, microtubules, and the plasma membrane [48]. Additionally, some DUBs are reported to function in more than one subcellular local, e.g., the cytosol and the nucleus, which most likely translocate following a specific cellular perturbation. Some achieve the correct locale by utilizing the internal localization signals such as the nuclear localization signal (NLS) or nuclear export signal (NES) that targets the protein in and out of the nucleus, respectively. Some translocate by utilizing the interaction with a partner protein, often by its non-catalytic domains or motifs, and some by PTMs.

The cytoplasmic DUB, USP10, is a novel regulator of p53 that counteracts Mdm2-induced p53 nuclear export and degradation. Following DNA damage or other genotoxic stress, a fraction of cytoplasmic USP10 is translocated into the nucleus, which is necessary for the activation and stabilization of p53. The translocation and stabilization of USP10 are controlled by ATM-dependent phosphorylation at Thr42 and Ser337 (Figure 3b). The expression of USP10 is downregulated in several cell carcinomas and the suppression of tumor cell growth with wild-type p53, suggesting that USP10 could function as a tumor suppressor [49]. However, the relationship between USP10 translocation and stabilization as well as how USP10 phosphorylation affects its stability and localization have not yet been understood. More evidence is needed to establish the physiological role of USP10 phosphorylation in tumorigenesis.

In the case of ATX3, phosphorylation at Ser340 and Ser352 by CK2 enhances its nuclear localization, aggregation, and stability, processes that play a major role in the development of spinocerebellar ataxia-3. The two residues are located in the third UIM of ATX3, and the mutation of these sites strongly abrogates the formation of nuclear inclusions. ATX3 interacts with CK2α, while the pharmacological inhibition of CK2 reduces the amount of nuclear ATX3 levels as well as inclusions formation [50]. Additionally, the protein kinase CK2- and GSK3β-mediated phosphorylation of ATX3 at the highly conserved Ser29, resides in the N-terminal Josephin domain, promotes ATX3 nuclear uptake. Ser29 phosphomutant of ATX3 showed a reduction in translocation efficiency to the nucleus [51]. ATX3 phosphorylation may target pathological ATX3 to the nucleus, where it eludes cytoplasmic ubiquitination and proteasomal degradation and forms nuclear aggregates [52]. Thus, pharmacological modulation of ATX3 subcellular distribution by phosphorylation may provide a reasonable therapeutic approach for SCA3.

Phosphorylation of OTUB1 by casein kinase 2 (CK2) causes nuclear translocation for DNA damage repair [53]. The AKT-mediated phosphorylation of USP4, on the other hand, triggers its subcellular localization predominantly in the membrane and cytoplasm, whereas the non-phosphorylated form is mostly condensed into the nucleus [54]. Both USP8 and CYLD undergo EGF-mediated translocation to the endosomes and associated with a phosphotyrosine protein interaction network [55,56]. However, in the case of USP8, phosphorylation at Ser680 is also critical for its subcellular localization since mutation of Ser680 to Ala restricts its localization to the nucleus, whereas the wild type is predominantly localized into the cytosol essential for USP8 interaction with the protein 14-3-3ε [57]. The ubiquitination of JosD1 triggers its localization to the membrane from the cytoskeletal fraction [38]. Membrane-associated farnesylation of UCHL1 was reported to promote α-synuclein accumulation, which is related to Parkinson’s disease [58] and has an important role in the transport of Epstein-Barr virus primary oncoprotein LMP1 to the exomes [59]. Although farnesylation is important in maintaining protein stability, it is not shown to be required for membrane association in primary neurons [60]. Lipid modification of USP32 is involved in its association with intracellular membranes [61].

### 2.3. PTMs Regulating DUBs Interaction with Partner Proteins

For some DUBs, interaction with a partner protein is crucial for their activity, i.e., interacting partner proteins affect DUB catalytic activity by assisting substrate recognition and specificity, or translocate a particular DUB to the right locale. Many DUBs have extra domains and motifs besides the catalytic domain, and these non-catalytic regions often participate in the interaction with other partner proteins and factors. For example, UIM is required for the efficient hydrolysis of USP25 [41] and OTUD5 [62]. The interaction of the two endosomal DUBs, AMSH, and USP8 (UBPY in yeast), with a UIM-containing signal transducing adaptor molecule 2 (STAM2) enhances their deubiquitinating efficacy via substrate arrest [63,64]. Additionally, two JAMM domain-containing proteins, PSMD14 (also known as Pad1, POH1, and Rpn11 in yeast) and COPS5 (also known as CSN5, JAB1, MOV-34, and SGN5), need to incorporate into higher-order protein structures such as 19S proteasome or COP9 signalosome, respectively, for their DUB activity [65,66]. However, some interacting proteins also inhibit DUB activity, e.g., the DUB activity of UCHL5 (also known as UCH37, CGI-70, and INO80R) is reduced when associated with the chromatin-remodeling complex INO80 [67].

In the case of USP1, the cofactor USP1-associated factor 1 (UAF1) is required for both the DUB activity and subcellular localization. The interaction with UAF1 is regulated by USP1 phosphorylation at Ser313 [68,69,70]. Importantly, the DUB activity of USP1 is enhanced by about 36-fold in the presence of UAF1 [71]. Once the USP1-UAF1 complex is formed, it translocates into the nucleus where the recruitment of FANCD2 and PCNA substrates using a SUMO-like domain takes a place to regulate DNA damage response [72]. Interestingly, USP12 and USP46 are activated by two β-propeller proteins, UAF1, and WDR20. The ternary complex crystal structures show that the two partner proteins stabilize the structural elements around the catalytic site of the DUBs, which consequently have a synergistic effect on the activation of each DUB [73,74,75]. WDR20 binding to USP12 and USP46 showed a significant increase in the catalytic activity in vitro [71,76]. Since USP1 shares high sequence homology with USP12, similar effects are expected. The interaction with UAF1 is regulated by USP1 phosphorylation at Ser313 [70].

Several DUBs are reported to function in more than one cellular compartment. For example, USP4 and USP15 function in both the nucleus and the cytosol. In the cytosol, USP4 deubiquitinates the member of many vital cell signaling pathways, e.g., NF-κB [77], TGF-β [54], Wnt/β-catenin [78], and p53 [79] as well as adenosine A2A receptor [80] and the E3 ligase TRIM21 [81]. Likewise, USP15 is also involved in many cellular pathways, such as neuroinflammation [82], T-cell activation [83], Nrf2-Keap1 pathway [84], and bone morphogenetic protein (BMP) signaling [85], as well as regulation of the COP9 signalosome [86] and TGF-β activity [87] in the cytosol. The two DUBs also regulate spliceosome dynamics by deubiquitinating the spliceosome proteins in the nucleus [88,89,90]. For both DUBs, the interaction with SART3 is essential for their nuclear translocation as well as co-localization to the substrates PRP31 and PRP3 required for regulating the spliceosome dynamics [89,90]. It should be noted that ^766^QPAKKKK^772^ of USP4 was identified as functional NLS [91]. A recent study showed that their cellular localization is further regulated by the phosphorylation of these two DUBs [92]. A study employing nuclear-cytoplasmic fractionation and mass spectrometric analysis showed that Thr149 and Thr219 of USP15, which is conserved among different species, are phosphorylated in the cytoplasm. These two sites are located at the UBL domain of USP15, which is important for the interaction with its partner protein SART3 for nuclear translocation (Figure 3c). The same applies to USP4 with Ser152 and Thr222 being the equivalent phosphorylation residues. These two paralogous DUBs share a strong structural homology and functional redundancy [93].

### 2.4. PTMs Regulating DUB Stability and Abundance

The stability and abundance of a particular protein are very important for modulating the biological functions and they are regulated by transcription, translation, and degradation. For DUBs, abundance and stability are also critical. For example, ATX3 phosphorylation at Ser256 by GSK3β regulates ATX3 aggregation [94]. Inhibition of S256 phosphorylation in normal ATX3 does not change its aggregation ability, but greatly increases its self-aggregation in expanded ATX3. S256A mutant of expanded ATX3 forms high molecular weight protein aggregation and the molecular chaperone Hsp70 represses the aggregation of S256A mutant, whereas S256D and expanded ATX3 without mutation on this site are predominantly monomeric.

Another DUB, USP7 also plays a critical role in maintaining genome stability and cancer prevention by regulating p53-Mdm2-related cellular networks. USP7 promotes deubiquitination and stabilization of mouse double minute 2 homolog (Mdm2) thereby enhances Mdm2-dependent p53 degradation. In addition to regulating other related proteins in this network, USP7 itself is a target for several PTMs. Phosphorylation of USP7 by CK2 at Ser18 stabilizes USP7, which in turn results in MDM2 stabilization and p53 downregulation. However, the phosphorylation is counterbalanced by ATM-dependent protein phosphatase PPMG1, and USP7 dephosphorylation causes MDM2 degradation and p53 stabilization (Figure 3d) [95]. USP7 is also phosphorylated at Ser963, but the functional consequence of this site has not yet been characterized in detail. Both phosphorylation sites are located near the regions that are involved in protein-protein interactions [96], suggesting they may play a critical role in interaction with the partner proteins. In addition to phosphorylation, USP7 is ubiquitinated at Lys869 by the HSV-1 regulatory protein ICP0, which has an E3 ligase activity as well. This Lys869 ubiquitination site is close to the ICP0 interaction region, which supports the proposition that USP7 is ubiquitinated by ICP0 but not by MDM2. ICP0 targets USP7 for proteasome-dependent degradation thus regulates its stability [97]. Challenges to inhibit USP7 activity and stability to increase p53 levels and induce apoptosis might be the new therapeutic perspective to develop anticancer drugs. USP10, another p53 targeting DUB that counteracts Mdm2-induced p53 nuclear export and degradation. CK2-mediated phosphorylation interacts and deubiquitinates only p53 but not the E3 ligase Mdm2. In USP10, phosphorylation at Thr42 and Ser337 by ATM kinase ensures its stability, which is critical for the activation of p53 [49]. Additionally, USP8 is stabilized by AKT-medicated phosphorylation at Thr907 [98]. Moreover, USP15 is phosphorylated during the cell cycle to regulate the mitotic degradation of the RE1-silencing transcription factor (REST) and subsequently dephosphorylated in early G1 while REST is stabilized and re-accumulated [99]. Furthermore, USP15 isoform-1 is phosphorylated at Ser229 residue during mitotic entry, which selectively abrogates the role of USP15 in maintaining TOP2A mediated genomic stability [100].

## 3. Misregulation of DUB PTM May Lead to Disease

**Figure 4:**
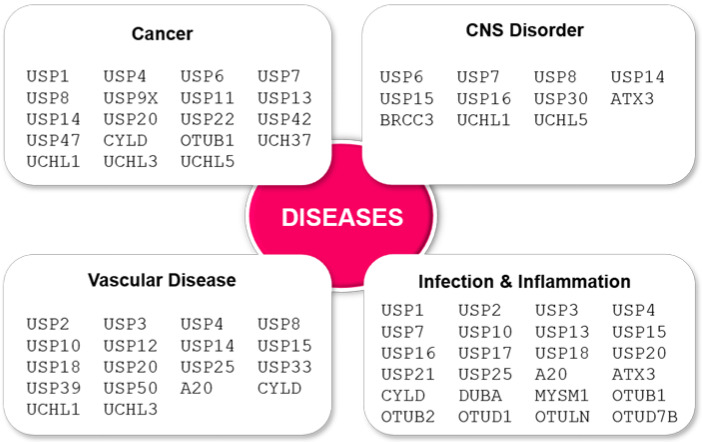
Disease-related DUBs.

Dysfunction of the deubiquitination system, as in ubiquitination, has been shown to lead to diseases such as cancer, neurological disorders, inflammation, infectious diseases, and cardiovascular diseases [114,115,116,117,118,119,120,121]. Figure 4 shows the DUBs that are associated with several diseases, including ones whose underlying mechanisms are not yet fully understood. Although the significance of PTM-mediated DUB function in disease is not quite clear, evidently, PTMs are an indispensable way to the functional regulation of DUBs associated with several diseases. The following are some well-known and extensively studied examples.

Phosphorylation of CYLD by IKKε promotes cell transformation by increasing NF-κB activation [104], but the presence of the IKK inhibitors leads to apoptosis indicating that CYLD may be a novel therapeutic target for adult T-cell leukemia [31]. A recent report on the varying degree of A20 phosphorylation (T108A/I207L > I325N > C243Y) at Ser381 by IKKβ showed a significant effect on immune response and microbial tolerance and resistance depending on the extent of phosphorylation. The rare allele C243Y, with almost 95% loss of A20 phosphorylation, causes a severe inflammatory disorder in both mice and humans [33]. USP7 is another well-known cancer-associated DUB that interacts with the tumor suppressor gene p53 and USP7 deubiquitinating function may protect cells from apoptosis. PTMs including phosphorylation and ubiquitination are reported to involve in USP7 [96], but the featured relevance of these modifications in its functional disorder has not yet been well understood. PTM of certain DUBs have also a vital role in a group of neurodegenerative diseases. For example, SCA3 is regulated by multiple PTMs of ATX3. Phosphorylations of ATX3 by protein casein kinase 2 (CK2) stimulate SCA3 pathogenesis by altering its stability, nuclear localization, and inclusion formation [50,51], while GSK3β-mediated phosphorylation inhibits ATX3 aggregation which has a protective role in SCA3 pathophysiology [94]. The DUB activity of ATX3 is also promoted by its ubiquitination regulating Ub-dependent homeostasis as well as neuroprotection in SCA3 by proteasomal degradation of misfolded proteins [109]. In addition, SUMOylation adjusts SAC3 pathogenesis by regulating ATXN3 stability and aggregation [122]. UCH-L1 is highly expressed in neurons and is assumed to involve in several neurodegenerative disorders including Parkinson’s disease. *O*-glycosylation of UCH-L1 in the nerve terminals [113] or monoubiquitination at multiple lysine residues within the active site control its enzymatic activity [34].

Since their discovery, DUBs were considered as a promising class for drug discovery across diverse therapeutic areas [119]. Much effort has been focused on developing small molecules targeting these DUBs modulating the activity (and/or localization), and some are getting ready for clinical trials [123,124,125,126]. However, the complexity of DUB regulation as well as the enzymatically abandoned or physiologically redundant nature of DUBs makes them a challenging target. As with kinases, there are certainly going to be DUBs for which inhibition needs to be avoided. Although it was not discussed in-depth, since the crosstalk between ubiquitination and phosphorylation, perhaps it may be possible to repurpose existing kinase inhibitors to modulate DUBs. Additionally, databases such as COSMIC (COSMIC catalog of somatic mutations in cancer; https://cancer.sanger.ac.uk/cosmic) or mapping PTM sites to proteins with available three-dimensional structural information might be useful tools [127]. Nevertheless, with a recent accumulation of the structural evidence, binding modes, and development in biochemical assays, more exciting activities in drug discovery are expected.

## 4. Conclusions

As seen throughout this review, DUBs are tightly regulated by PTMs for their activity, localization, stability, and interactions. In addition, certain PTMs requires another PTM, while two PTMs can compete for the same site, adding yet another layer of complexity. Since ubiquitination is one of the most important PTMs, often essential for cell viability, deubiquitination is equally important and requires tight regulation. Thus, DUB PTMs can serve as a feedback mechanism to ensure the proper function of DUBs. As the relevance of DUBs in different human diseases accumulates, the PTM-medicated regulation of DUBs should be examined more closely. DUB itself might not be the significant disease driver in some cases, but modulation of DUB function may provide a critical cause for different diseases. Hopefully, recent advancements in technologies and other tools, including bioinformatics workflow will allow the detection of not only novel PTMs but also the known PTMs on previously unrecognized modified proteins. A better understanding of PTM-mediated regulation of DUBs might provide us yet new insight to overcome a disease.

## Figures and Tables

**Table 1:** Known PTMs of various DUBs. Listed are the names of DUB, modified sites, effect in the cells according to PTM, as well as important references.

DUBs	Modified Sites	Effect in Cells	References
***Phosphorylation***			
USP1	Ser313	Promote DUB catalytic activity by enhancing the interaction with cofactor UFA1	[70]
USP4	Ser445	Alter the subcellular localization from the nucleus to cytoplasm	[54]
USP7	Ser18	Stabilization of USP7 for MDM2 deubiquitination	[95]
	Ser963	Unknown (possibly protein-protein interaction)	[96]
USP8	Ser680	Inhibit catalytic activity by promoting USP8 association with the 14-3-3 family proteins	[20]
	Tyr717, Tyr810	Elevates activity leading to inhibition of ciliogenesis	[24]
	Thr907	Increase Stability	[101]
USP9X	Ser1600	Enhance catalytic activity	[25]
USP10	Thr42, Ser337	Increase stability by inducing nuclear translocation	[49]
USP14	Ser432	Increase catalytic activity	[15]
USP15	Thr149, Thr219	Regulate interaction, localization and DUB activity towards its substrate PRP31	[92]
	Ser229	Abrogate USP15 function in maintaining TOP2A mediated genomic stability	[100]
USP25	Tyr740	Decrease USP25 cellular level and stability	[102]
USP28	Ser67, Ser714	Regulate the complex-formation with the DNA checkpoint proteins	[103]
USP37	Ser628	Enhance catalytic activity in G1/S during cell cycle.	[17]
USP44	Unknown	Activation of USP44	[18]
CYLD	Ser418	Decrease DUB activity and induce IKKε-mediated cell transformation	[104]
OTUD5	Ser177	Required for activation of the enzyme	[27]
A20	Ser381	Increase the activity of A20 to inhibit NF-κB signaling pathway	[32]
ATX3	Ser340, Ser352	Increase nuclear localization and aggregation	[50]
	Ser129	Promote nuclear uptake	[51]
OTUB1	Ser16, Ser18, Tyr26	Increase stability and protein–protein interaction	[105]
BAP1	Thr273, Ser276, Ser592	Promote DNA repair and cellular recovery from DNA damage	[106]
***Ubiquitination***			
USP6	Mono-ubiquitination	Promotes its own deubiquitination	[107]
USP7	Lys869	Decrease stability and modulate protein-protein interaction	[96]
USP25	Mono-ubiquitination at Lys99	Enhance catalytic activity and substrate recognition	[41]
USP30	Fingers subdomain of the catalytic domain	Induce proteasomal degradation	[108]
USP44	Unknown	Diminish stability by inducing proteasomal degradation.	[19]
ATXN3	Lys117	Enhance catalytic activity	[109]
JosD1	Unknown	Enhance catalytic activity and regulates membrane dynamics, cell motility, and endocytosis	[38]
UCHL1	Lys4, Lys65, Lys71, Lys157	Inhibit enzyme activity	[34]
BAP1	Multiple mono-ubiquitination within the NLS region (residues 699–729)	Prevent cytoplasmic accumulation by auto deubiquitination	[110]
***SUMOylation***			
USP25	Lys99, Lys 141	Inhibit catalytic activity by decreasing chain hydrolysis	[39]
USP28	Unknown	Negatively regulate the deubiquitinating activity	[40]
CLYD	Unknown	Inhibit activity against TRAF2 and TRAF6 and facilitates NFκB signaling	[111]
ATXN3	Lys166	Enhance stability	[112]
***Others***			
USP1	Oxidation	Reduce catalytic activity	[45]
USP32	Lipid modification	Association with intracellular membranes	[11]
UCHL1	Farnesylation	Promote intracellular membrane association and increased α-synuclein accumulation	[58]
UCHL1	O-glycosylation	Regulate synaptosome proteins functions playing a vital role in neurodegenerative disease.	[113]

## Abbreviations

PTM	post translational modification
DUB	deubiquitinating enzyme
Ub	ubiquitin
USP	ubiquitin-specific protease
JAMM	JAB1/MPN/Mov34 metalloenzymes
OTU	ovarian tumor protease
UCH	ubiquitin C-terminal hydrolase
MJP	Josephin and JAB1/MPN^+^
MINDY	MIU-containing novel DUB
ZUP1	zinc finger-containing ubiquitin peptidase 1
SUMO	small ubiquitin-like modifier
UBA	ubiquitin associated domain
UBL	ubiquitin-like
UIM	ubiquitin interacting motif
MJD	Machado-Josephin domain protease
